# The role of repetitive transcranial magnetic stimulation therapy in functional bowel disease

**DOI:** 10.3389/fmed.2023.1249672

**Published:** 2023-12-22

**Authors:** Guangyao Li, Tingcong Lv, Binghui Jin, Zhe Fan

**Affiliations:** ^1^Department of General Surgery, The Third People’s Hospital of Dalian, Dalian Medical University, Dalian, China; ^2^Department of Central Laboratory, The Third People’s Hospital of Dalian, Dalian Medical University, Dalian, China; ^3^Liaoning Province Key Laboratory of Corneal and Ocular Surface Diseases Research, The Third People’s Hospital of Dalian, Dalian Medical University, Dalian, China; ^4^Department of Oncology, Cancer Hospital of Dalian University of Technology, Shenyang, China

**Keywords:** functional bowel disease, biophysical therapy, repetitive transcranial magnetic stimulation, brain-gut axis, anxiety, depression

## Abstract

**Objective:**

This study investigates the effectiveness of repetitive transcranial magnetic stimulation (rTMS) as a biophysical therapy for alleviating symptoms of functional bowel disorder (FBD) and associated psychological symptoms by targeting the brain-gut axis.

**Methods:**

We conducted a comparative analysis involving 226 subjects, comprising the FBD group (*n* = 113) and a healthy control group (*n* = 113). Within the FBD group, participants were further divided into those who received rTMS therapy (FBD treatment group, *n* = 63) and those who did not (FBD control group, *n* = 50). The FBD treatment group was subcategorized based on the number of rTMS treatments received. We evaluated various factors, including gender, age, monthly household income, daily activity level, and sleep quality, as potential risk factors for FBD. Severity assessments of FBD and associated symptoms (constipation, anxiety, depression, and somatization disorders) were conducted using validated scales before and after treatment.

**Results:**

Our findings revealed a higher incidence of FBD in women, with most cases emerging at age 50 or older. We identified lower monthly household income, reduced daily activity levels, and poorer sleep quality as factors associated with a higher likelihood of FBD. FBD patients exhibited higher scores for constipation, anxiety, depression, and somatization disorders compared to healthy controls. rTMS therapy was effective in reducing gastrointestinal symptoms, anxiety, depression, and somatization disorders among FBD patients. Notably, the extent of improvement was positively correlated with the number of rTMS sessions. No adverse effects were observed during the study.

**Conclusion:**

Our study underscores the efficacy of biophysical therapy, specifically repetitive transcranial magnetic stimulation, in mitigating FBD symptoms and associated psychological distress. The treatment’s effectiveness is positively linked to the frequency of rTMS sessions.

## 1 Introduction

Functional Bowel Disorder (FBD) is a widespread gastrointestinal condition that imposes a substantial burden on global healthcare systems and diminishes patients’ quality of life (1). It encompasses a group of intestinal disorders characterized by recurrent symptoms, but without identifiable physical causes, such as organic lesions (2, 3). The exact causes of FBD are not fully understood, but they likely result from a complex interplay of physiological, psychological, genetic, social, and early-life factors (4). Over time, persistent gastrointestinal symptoms often lead to mental health issues, including anxiety and depression (5). These long-term psychiatric symptoms can impact various bodily systems, including the hypothalamus and autonomic nerves, thereby worsening intestinal discomfort and increasing the treatment burden (6).

Rome IV criteria have recognized the brain-gut axis as a fundamental element in the pathophysiology of functional bowel disease (7). A growing body of evidence supports the idea that functional bowel disease evolves through three distinct phases: Stage I, characterized by gastrointestinal motility disorder; Stage II, marked by visceral hypersensitivity; and Stage III, featuring bidirectional dysfunction of the brain-gut axis (8, 9). The brain-gut axis represents a two-way regulatory network connecting the brain and the gastrointestinal tract through a complex neuro-immune-endocrine network, with the central, autonomic, and enteric nervous systems playing key roles (10, 11). In this model, emotions, thoughts, and perceptions influence various aspects of gastrointestinal function, including sensation, secretion, motility, immune regulation, mucosal inflammation, and permeability (12). Conversely, alterations in gastrointestinal function can affect conscious perception and behavior in the brain (4). Communication between the brain and the enteric nervous system occurs through the autonomic nervous system and the hypothalamic–pituitary–adrenal axis, enabling stressors in the brain to impact gut function and vice versa. This bidirectional signaling may play a crucial role in the pathophysiology of functional bowel disease (13).

Transcranial magnetic stimulation (TMS) emerges as a non-invasive technique for brain stimulation. TMS generates brief, rapidly changing magnetic fields that induce electrical currents in the brain, with a high degree of penetration into the brain tissue (14). It is utilized in various modes, including single-pulse and double-pulse TMS for exploring brain function and repetitive transcranial magnetic stimulation (rTMS) for inducing lasting changes in brain activity beyond the stimulation period (15). The effects of rTMS depend on the intensity and frequency of stimulation, with low-frequency (<1 Hz) rTMS having inhibitory effects and high-frequency (>5 Hz) rTMS producing excitatory effects (16). Despite being relatively new in development, TMS has shown significant therapeutic potential in various clinical areas, offering promising avenues for studying brain function. In medical treatment, TMS has been widely used for epilepsy (17), Parkinson’s (18), depression (19), neuropathic pain (20), stroke (21), diabetic neuropathy (22), multiple sclerosis (23), tinnitus (24), eating disorders (25), addiction (26), and obsessive-compulsive disorder (27). In China, an expert consensus has recommended rTMS clinical treatment options for a range of conditions, including depression, pain, movement disorders, stroke, epilepsy, tinnitus, anxiety disorders, obsessive-compulsive disorder, schizophrenia, substance addiction, and sleep disorders, based on clinical studies published as of November 2017 and evidence-based medical standards (28). However, its application to functional bowel disease remains relatively uncharted territory.

## 2 Subjects and methods

### 2.1 Participants and groups

This study was a retrospective study. The COVID-19 pandemic significantly disrupted healthcare systems and patient hospitalization patterns. As a result, the number of available patients for our study was substantially reduced, especially for the period following the pandemic’s onset. Given these constraints, we decided to focus exclusively on patient information collected before the arrival of COVID-19. A total of 113 people diagnosed with FBD were selected as the FBD group. Patients with FBD who attended the outpatient clinic of Dalian Third People’s Hospital from March 2019 to December 2020 and met the diagnostic criteria for Rome IV (29) were selected. Patients who attended the outpatient clinic for physical examination during the same period were selected as healthy controls in a ratio of 1:1, with a total of 113 cases. Among the above 113 patients with FBD, 63 patients with rTMS treatment were selected as the FBD treatment group, and the remaining 50 untreated patients were selected as the FBD control group. For categorization purposes, the FBD treatment group was stratified according to the cumulative number of rTMS sessions. Specifically, the group labeled as FBD treatment group (rTMS <200 sessions) included participants who underwent fewer than 200 rTMS sessions over the four-week treatment duration, whereas the group labeled as FBD treatment group (rTMS ≥200 sessions) consisted of participants who completed 200 or more rTMS sessions within the same period (Table 1).

**Table 1:** Distribution of patients receiving different number of treatments.

Treatment sessions range	Number of patients
10–49	2
50–99	8
100–149	14
150–199	8
200–249	16
250–299	5
300–349	0
350–399	2
400–450	8

This study has been reviewed by the Ethics Committee of Dalian Third People’s Hospital. All selected patients have fully understood the purpose and process of this experiment and signed an informed consent form.

### 2.2 Questionnaires

All selected individuals were asked to complete the General Information Questionnaire, the Constipation Severity Scale (CSS), the Self-rating Anxiety Scale (SAS), the Self-rating Depression Scale (SDS), and the Somatization Symptom Scale (SSS). The FBD group was required to fill out the above scale before and after treatment, while the healthy control group only needed to fill out one form during the physical examination.

A self-administered general information questionnaire was used to collect social information about the enrolled individuals, including name, gender, age, occupation, marital status, family relationship, monthly household income, education, diet and sleep status, history of smoking and drinking, exercise, and past medical history.

The CSS (30) is a constipation score developed by the Cleveland Clinic. There are 8 self-test questions, with a full score of 30 points, and a minimum of 0 points. The higher the score, the more serious the degree of constipation.

The SAS (31) was developed by the Duke University School of Medicine and is widely used for screening and diagnosing anxiety symptoms in psychiatry and counseling. There are 20 self-test questions, including 15 positive and 5 negative scoring questions, which are based on how the person has been feeling for the last week. Each question is divided into 4 levels and scored according to 1, 2, 3, 4. A standard score of 50 was used as the threshold, with a standard score of less than 50 being no anxiety and a standard score of greater than or equal to 50 being an anxiety state.

The SDS (31) is one of the scales recommended by the U.S. Department of Education and Welfare for use in psychiatric research and provides a simple and intuitive response to the subjective feelings of depressed patients. The scale consists of 20 self-administered questions, including 10 positive and 10 negative scoring questions. The standard reference value is 53, less than 53 is no depression, and greater than or equal to 53 is a depressive state.

The SSS (32) was developed by Professor Mao of Shanghai Jiaotong University according to the characteristics of somatic symptoms as the main manifestation of psychological disorders in patients in general hospitals. The scale consists of 20 self-test questions, and each question is divided into four levels according to the severity of symptoms and scored according to 1, 2, 3, 4. The standard reference score is 36, and 36 scores of and above are classified as mild to moderate severity.

To ensure the accuracy and validity of the scale scores, all self-assessment scales were completed by the enrolled participants under the guidance of the investigator. The site environment was quiet and free of distracting factors. After the scales were completed, they will be taken back on the spot. If there was any doubt about the content of the scales, the researcher explained it to them in time to ensure the authenticity and reliability of the scale scores.

### 2.3 Repetitive transcranial magnetic stimulation and treatment programs

Our study utilized the YRD CCY-I magnetic field stimulator produced by Wuhan YIRUIDE Medical Equipment New Technology Co., Ltd. The FBD treatment group consisted of 63 patients who received treatment using 1 Hz rTMS stimulation. The FBD control group of 50 patients received sham rTMS treatment without current. The right dorsolateral prefrontal cortex (DLPFC) was selected as the stimulation site (33). The stimulation frequency was set to 1 Hz, with the stimulation parameters adjusted to 80% of the motor threshold (34). The motor threshold was determined using the conventional method of measuring the minimum intensity required to induce a visible twitch in the contralateral thumb muscle (35). This method is widely accepted and was used to ensure the safety and efficacy of the rTMS sessions. The duration of stimulation was established at 8 s, with a total of 8 pulses delivered during each session. The inter-pulse interval was fixed at 3 s. The total treatment duration for each session was 15 min. Each patient performed 20 rTMS sessions per day, waiting 2 min between each rTMS session to ensure the coils were cool (36). The treatment protocol consisted of 5 days of stimulation, followed by a rest period of 2 days. The overall treatment cycle spanned 4 weeks, resulting in a cumulative treatment duration of 20 days. The room was equipped only with a magnetic field stimulator and necessary computer equipment, without any other electronic devices or instruments. A comfortable treatment chair was provided for the patients. Patients were asked to sit comfortably in the chair, relax their entire body, and keep their head as still as possible. Prior to treatment, all metallic objects that could interfere with the instrument were removed from the patient’s body. During the treatment, the instrument produced a certain amount of noise, which could cause discomfort to the patient. Therefore, patients were provided with headphones to avoid interference. The lighting in the treatment room was dimmed, and the room was relatively quiet, providing a comfortable environment for patients during the treatment.

### 2.4 Sham repetitive transcranial magnetic stimulation

To maintain consistency, the sham rTMS coil is positioned over the same scalp location as the active treatment group, with an identical coil orientation replicating the physical sensation of the magnetic pulse while withholding the therapeutic stimulation. The intensity of the sham rTMS is carefully adjusted to remain below the motor threshold, ensuring that participants can feel the coil’s presence on their scalp without receiving the actual therapeutic treatment. The pulse sequence used for sham rTMS is designed to mimic the sensory experience of real rTMS pulses, including both sound and sensation (37). These sham pulses are delivered in a randomized pattern to prevent participants from discerning any patterns associated with the active treatment. To mitigate potential bias, “double-blind” conditions are upheld, wherein both participants and the researchers conducting the rTMS sessions remain unaware of the treatment allocation. Throughout the study, continuous data collection is performed to monitor participants’ experiences and perceptions during sham rTMS sessions, confirming that the sensations closely resemble those induced by real rTMS.

### 2.5 Assessment of side effects

The safety and well-being of participants were of utmost importance in this study. As such, we closely monitored for potential side effects associated with TMS. The most common side effects of TMS include headache, scalp discomfort, lightheadedness, and a tingling sensation at the treatment site (38). More serious side effects, such as seizures, are extremely rare but were also monitored.

To assess the presence and severity of side effects, participants were asked to report any adverse experiences immediately following each TMS session. Additionally, a standardized side effect questionnaire was administered to all participants at the beginning and end of the treatment period. This questionnaire included items related to headache, scalp discomfort, lightheadedness, and other common side effects of TMS.

To minimize the occurrence of side effects, we followed established safety guidelines for the administration of TMS, including individualized determination of the motor threshold to ensure appropriate stimulation intensity and careful placement of the TMS coil to target the desired brain region accurately. Furthermore, participants were given a thorough explanation of the procedure and potential side effects before commencing the study, and they were encouraged to report any discomfort or adverse experiences promptly.

### 2.6 Pre-experiment preparation

Both the FBD group and the healthy controls were required to discontinue any medication that could potentially influence the study outcomes. Participants in the FBD group should complete relevant tests (ECG, routine blood test, routine urine test, routine stool test, occult blood test, liver function, kidney function, etc.) before the start of the experiment.

### 2.7 Statistical analyses

All statistical analyses were performed using the statistical analysis software SPSS (version 26.0, SPSS Inc., Chicago, IL, USA). Measurement data conforming to normal distribution were expressed as mean ± SD. Independent samples *t*-tests were used for comparison between groups, and paired *t*-tests were used for self-control before and after. The measurement data did not conform to a normal distribution using the rank sum test, expressed as M (P25, P75). Enumeration data were analyzed by *χ*^2^ test and expressed as frequency/percentage. A value of *p* < 0.05 was considered statistically significant.

## 3 Results

### 3.1 General situation analysis of the FBD group

**Figure 1:**
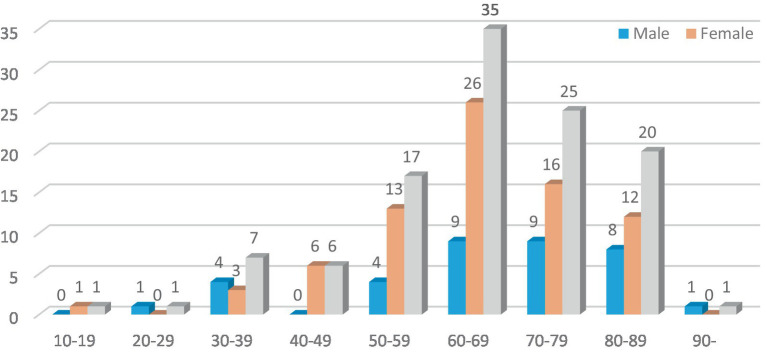
Distribution of female and male of the FBD group.

In this study, there were 113 patients in the FBD group, and the incidence of female patients was significantly higher than that of male patients, including 36 male patients (31.86%) and 77 female patients (68.14%). The average age was 65.45 ± 14.64 years old. Taking 10 years as an age group, the age of onset of patients in the FBD group was mainly concentrated in the age group of 50 years or older, among which the age group of female patients was mainly concentrated in the age group of 60–69 years, and the age group of male patients was mainly concentrated in the age group of 60–79 years (Figure 1).

**Figure 2:**
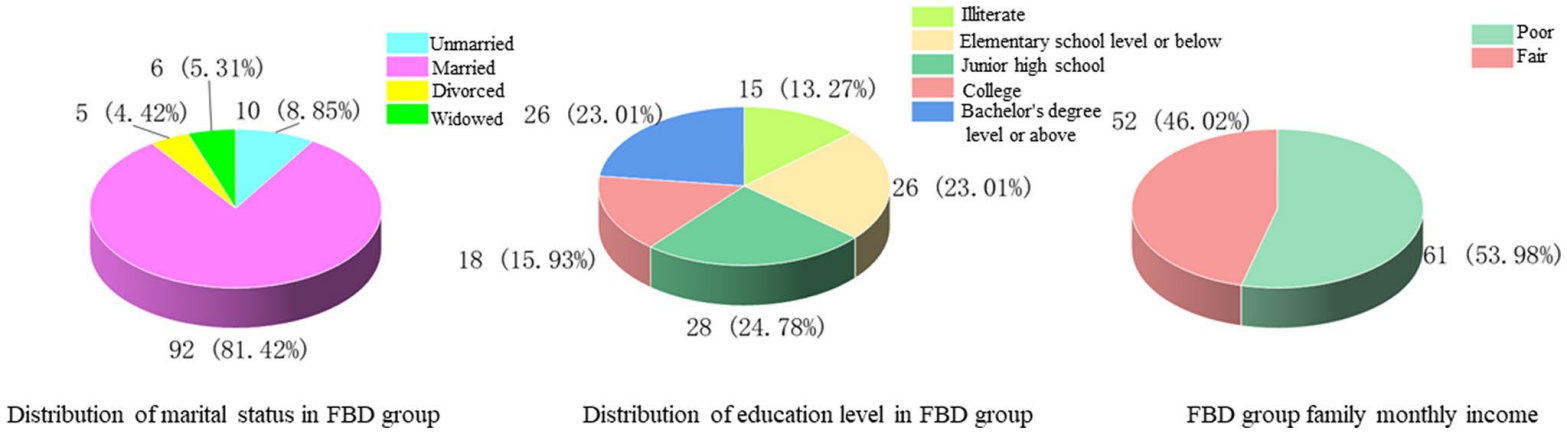
Marital status, educational attainment, and monthly household income of the FBD group.

Among the marital status, 10 cases (8.85%) were unmarried, 92 cases (81.42%) were married, 5 cases (4.42%) were divorced, and 6 cases (5.31%) were widowed. Among the levels of education received, 15 cases (13.27%) were illiterate, 26 cases (23.01%) were educated at the elementary school level or below, 28 cases (24.78%) were at junior high school, 18 cases (15.93%) were at college, and 26 cases (23.01%) were at the bachelor’s degree level or above. In the comparison of monthly household income, the majority of cases [61 cases (53.98%)] had poor monthly household income, less than 5,000 yuan, and 52 cases (46.02%) had fair monthly household income, more than 5,000 yuan (Figure 2).

**Figure 3:**
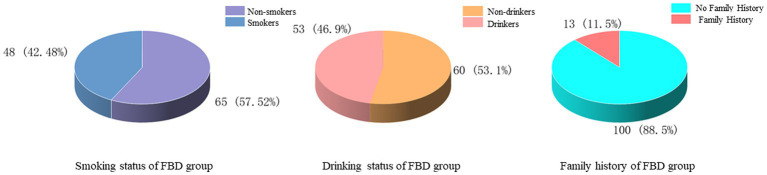
Smoking, alcohol consumption, and family history of the FBD group.

**Figure 4:**
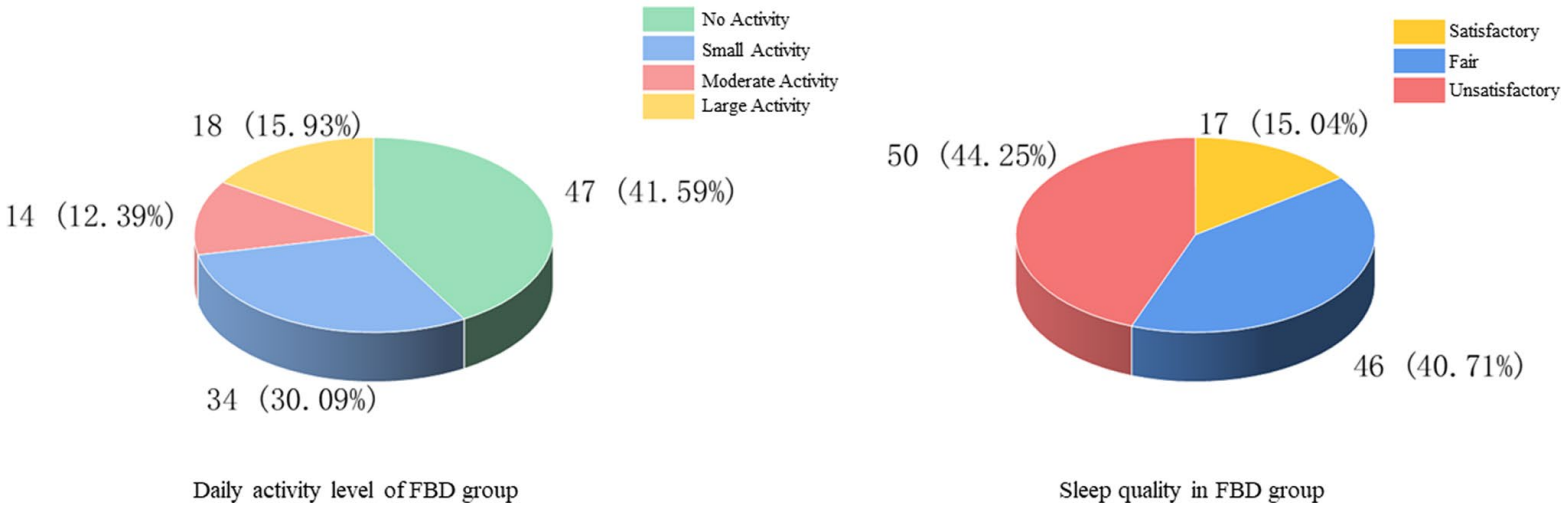
Daily activity and sleep quality of the FBD group.

The history of smoking and alcohol consumption was found in 48 (42.48%) and 53 (46.90%) cases, while 65 (57.52%) and 60 (53.10%) cases were non-smokers and drinkers. In 100 cases (88.50%), no previous family history was found, and 13 cases (11.50%) had family history (Figure 3). In the statistics of daily activity, 47 cases (41.59%) had no daily activity, 34 cases (30.09%) had a small amount of activity, 14 cases (12.39%) had moderate activity, and 18 cases (15.93%) had a large amount of daily activity. The quality of sleep was satisfactory in 17 cases (15.04%), fair in 46 cases (40.71%), and unsatisfactory in 50 cases (44.25%) (Figure 4).

### 3.2 General situation analysis of healthy control group

**Figure 5:**
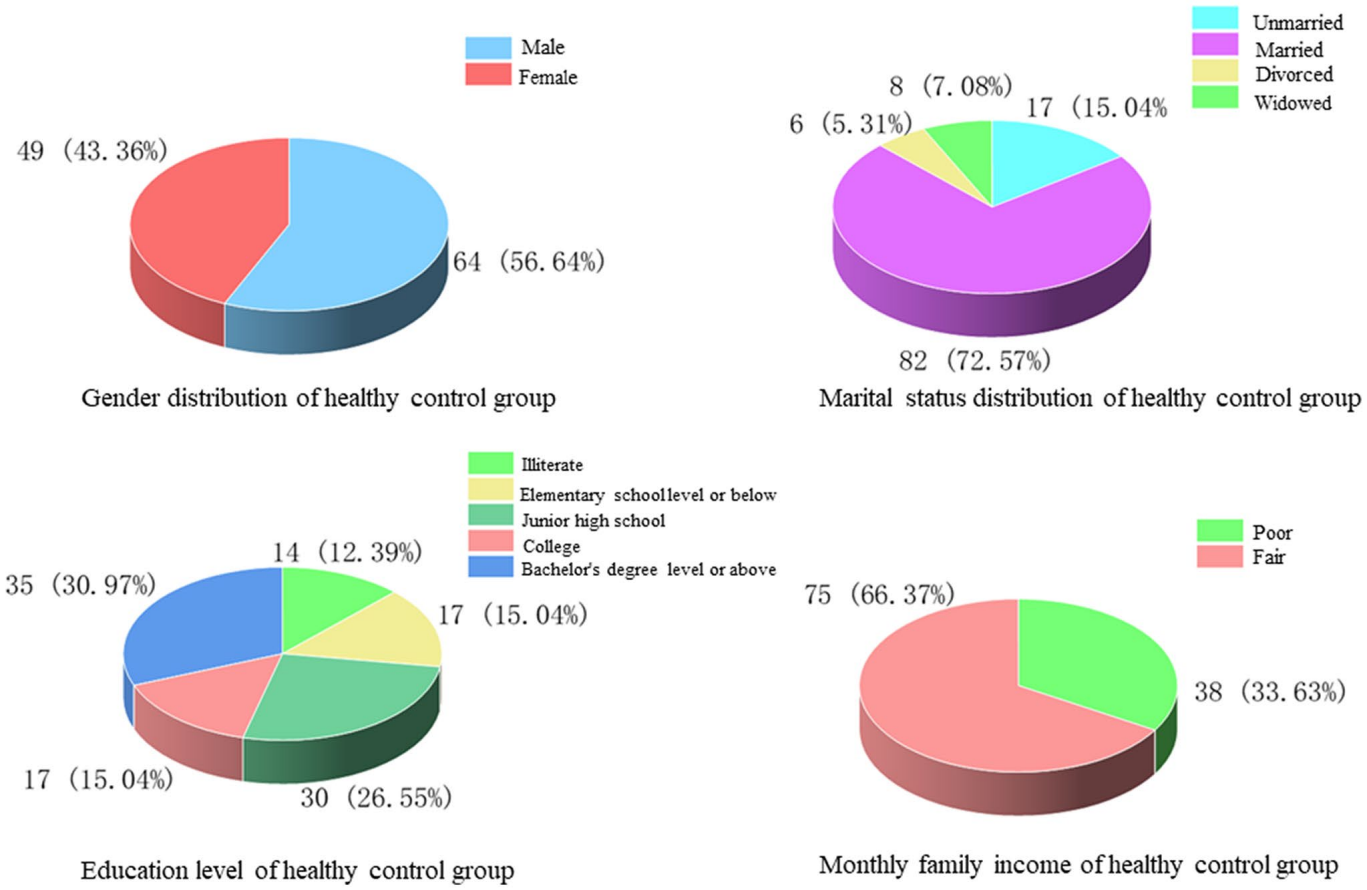
Sex, marital status, education and monthly household income of healthy control group.

In this study, 113 patients who attended the outpatient clinic for physical examination during the same period were selected as healthy controls, of whom 64 (56.64%) were male and 49 (43.36%) were female, with an average age of 47.12 ± 18.64 years. Among the marital status, 17 cases (15.04%) were unmarried, 82 cases (72.57%) were married, 6 cases (5.31%) were divorced, and 8 cases (7.08%) were widowed. Regarding the level of education received, 14 cases (12.39%) had no education, 17 cases (15.04%) were educated at elementary school and below, 30 cases (26.55%) at junior high school, 17 cases (15.04%) at college, and 35 cases (30.97%) at bachelor’s degree and above. The comparison of monthly household income showed that there were 38 cases (33.63%) with poor monthly household income and 75 cases (66.37%) with fair monthly household income (Figure 5).

**Figure 6:**
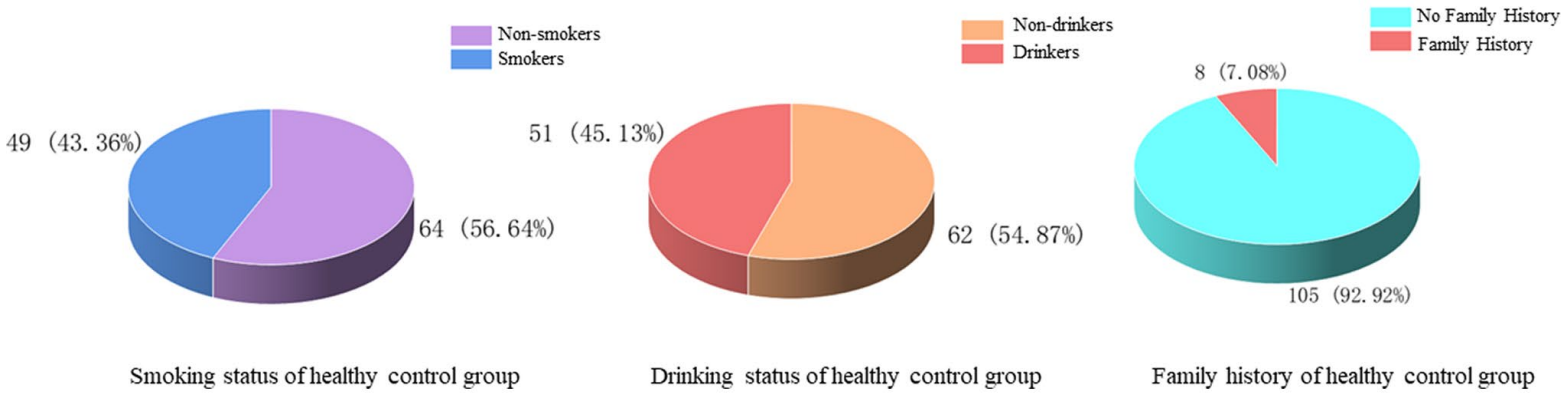
Smoking, alcohol consumption and family history of healthy controls.

**Figure 7:**
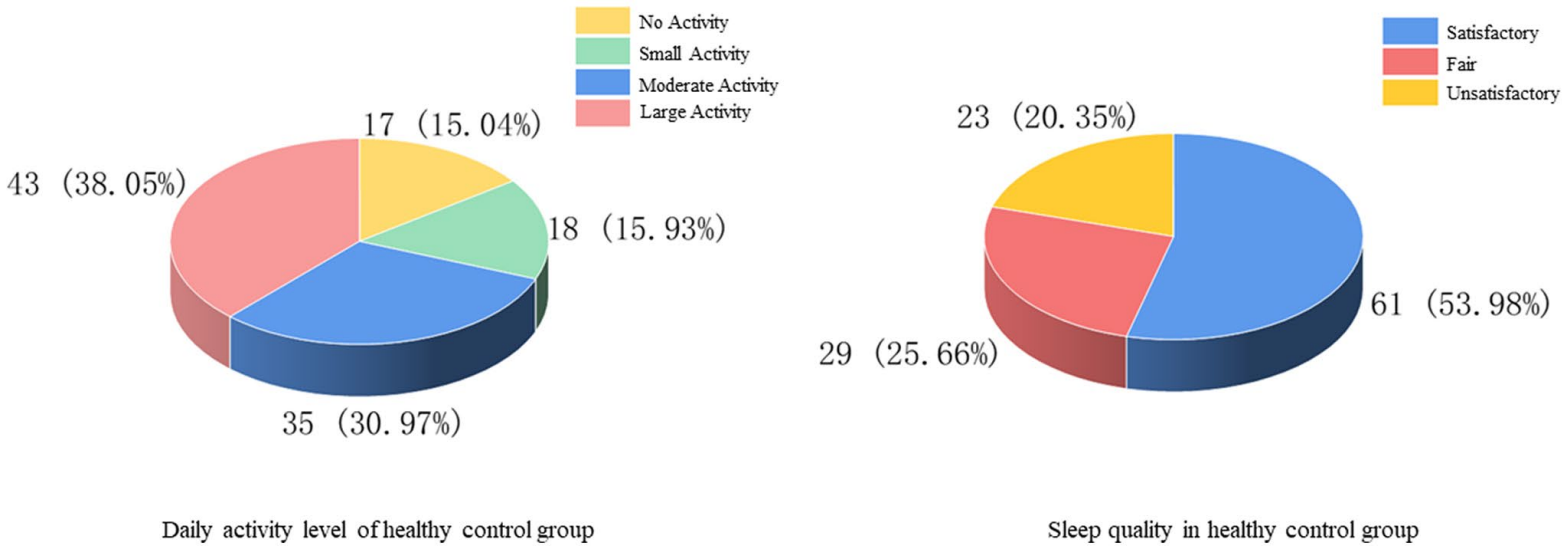
Daily activity and sleep quality of healthy control group.

There were 46 (40.71%) and 51 (45.13%) cases with a history of smoking and alcohol consumption, while 67 (59.29%) and 62 (54.87%) cases were non-smokers and drinkers. There was no previous family history in 105 cases (92.92%) and family history in 8 cases (7.08%) (Figure 6). In the statistics of daily activity, 17 cases (15.04%) had no daily activity, 18 cases (15.93%) had a small amount of activity, 35 cases (30.97%) had moderate activity, and 43 cases (38.05%) had a large amount of daily activity. The quality of sleep was satisfactory in 61 cases (53.98%), fair in 29 cases (25.66%), and unsatisfactory in 23 cases (20.35%) (Figure 7).

### 3.3 Comparative analysis of general conditions between the FBD group and healthy control group

Comparing the age of the FBD group with that of the healthy control group in the outpatient physical examination revealed no significant difference and was not statistically significant (*t* = 1.530, *p* = 0.127). For the comparison of marital status, no statistically significant difference was found between the two groups (*χ*^2^ = 2.766, *p* = 0.429). There was no statistically significant difference in the literacy level of the FBD group compared to the healthy control group (*χ*^2^ = 3.344, *p* = 0.502). The percentage of poor monthly household income was higher in the FBD group than in the healthy control group, which was statistically different (*χ*^2^ = 9.509, *p* = 0.002). The comparison between the two groups in terms of smoking (*χ*^2^ = 0.073, *p* = 0.787), alcohol consumption (*χ*^2^ = 0.071, *p* = 0.790), and family history (*χ*^2^ = 1.312, *p* = 0.252) revealed no significant difference between the two groups. The percentage of the FBD group with less daily activity was significantly higher than that of the healthy control group, which was statistically different (*χ*^2^ = 38.231, *p* < 0.001). The comparison in terms of sleep quality revealed that the percentage of poorer sleep quality was significantly higher in the FBD group than in the healthy control group, which was statistically different (*χ*^2^ = 38.660, *p* < 0.001) (Table 2).

**Table 2:** Comparative analysis of general conditions between the FBD group and healthy control group.

	FBD group (*n* = 113)	Healthy control group (*n* = 113)	t/*χ*^2^ value	*p* value
Age (years)	65.45 ± 14.64	62.50 ± 14.41	1.530	0.127
Gender			–	–
Male	36 (31.86%)	64 (56.64%)		
Female	77 (68.14%)	49 (43.36%)		
Marital status			2.766	0.429
Unmarried	10 (8.85%)	17 (15.04%)		
Married	92 (81.42%)	82 (72.57%)		
Divorced	5 (4.42%)	6 (5.31%)		
Widowed	6 (5.31%)	8 (7.08%)		
Education level			3.344	0.502
Illiterate	15 (13.27%)	14 (12.39%)		
Primary school and below	26 (23.01%)	17 (15.04%)		
Junior and senior high school	28 (24.78%)	30 (26.55%)		
Junior college	18 (15.93%)	17 (15.04%)		
Bachelor degree or above	26 (23.01%)	35 (30.97%)		
Monthly household income			9.509	0.002
Poor	61 (53.98%)	38 (33.63%)		
Satisfactory	52 (46.02%)	75 (66.37%)		
Smoking history			0.073	0.787
No	65 (57.52%)	67 (59.29%)		
Yes	48 (42.48%)	46 (40.71%)		
Drinking history			0.071	0.790
No	60 (53.10%)	62 (54.87%)		
Yes	53 (46.90%)	51 (45.13%)		
Family history			1.312	0.252
No	100 (88.50%)	105 (92.92%)		
Yes	13 (11.50%)	8 (7.08%)		
Daily activity			38.231	<0.001
No	47 (41.59%)	17 (15.04%)		
A small amount	34 (30.09%)	18 (15.93%)		
Moderate	14 (12.39%)	35 (30.97%)		
Massive	18 (15.93%)	43 (38.05%)		
Sleep quality			38.660	<0.001
Satisfactory	17 (15.04%)	61 (53.98%)		
Poor	46 (40.71%)	29 (25.66%)		
Unsatisfactory	5 0 (44.25%)	23 (20.35%)		

### 3.4 Analysis of CSS, SAS, SDS, and SSS scale scores in the FBD group and healthy control group before treatment

Comparing the pre-treatment scale scores of the FBD group with those of the healthy control group, it was found that the CSS scale score was 19.64 ± 2.57 in the FBD group and 8.24 ± 2.39 in the healthy control group. The CSS scale scores were significantly higher in the FBD group than in the healthy control group. There was a statistically significant difference in the overall mean CSS scale scores between the two groups (difference 11.398, 95% CI 10.747–12.049, *t* = 34.510, *p* < 0.001).

The SAS scale score was 65.70 ± 9.10 in the FBD group and 40.60 ± 9.68 in the healthy control group. The SAS scale scores were significantly higher in the FBD group than in the healthy control group. There was a statistically significant difference in the overall mean SAS scale scores between the two groups (difference 25.097, 95% CI 22.635–27.559, *t* = 20.088, *p* < 0.001).

The SDS scale score was 66.58 ± 9.05 in the FBD group and 44.38 ± 8.53 in the healthy control group. The SDS scale scores were significantly higher in the FBD group than in the healthy control group. There was a statistically significant difference in the overall mean SDS scale scores between the two groups (difference 22.204, 95% CI 219.898–24.509, *t* = 18.977, *p* < 0.001).

**Figure 8:**
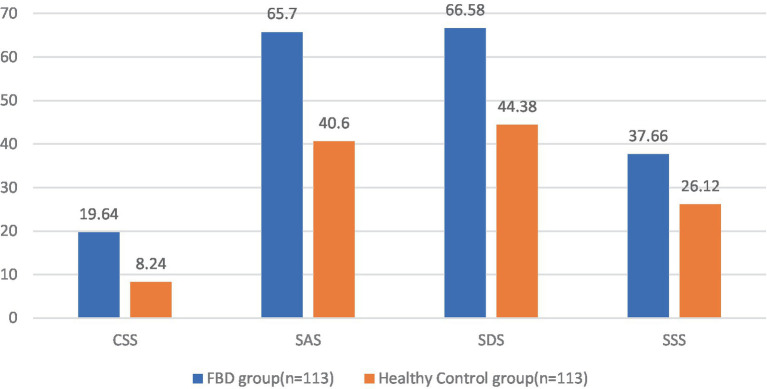
Comparison of scores between FBD group and healthy control group.

The SSS scale score was 37.66 ± 6.68 in the FBD group and 26.12 ± 6.36 in the healthy control group. The SSS scale scores were significantly higher in the FBD group than in the healthy control group. There was a statistically significant difference in the overall mean SSS scale scores between the two groups (difference 11.549, 95% CI 9.838–13.259, *t* = 13.307, *p* < 0.001) (Table 3 and Figure 8).

**Table 3:** Analysis of CSS, SAS, SDS, and SSS scale scores in the FBD group and healthy control group before treatment.

	FBD group (*n* = 113)	Healthy control group (*n* = 113)	Difference and 95% CI	*t* value	*p* value
CSS	19.64 ± 2.57	8.24 ± 2.39	11.398 (10.747–12.049)	34.510	<0.001
SAS	65.70 ± 9.10	40.60 ± 9.68	25.097 (22.635–27.559)	20.088	<0.001
SDS	66.58 ± 9.05	44.38 ± 8.53	22.204 (19.898–24.509)	18.977	<0.001
SSS	37.66 ± 6.68	26.12 ± 6.36	11.549 (9.838–13.259)	13.307	<0.001

### 3.5 Comparison of anxiety, depression, and somatization disorder between the FBD group and healthy control group before treatment

In the FBD group compared to the healthy control group, no anxiety was detected in 11 (9.73%) and 89 (78.76%) cases, mild anxiety in 17 (15.04%) and 17 (15.04%) cases, moderate anxiety in 47 (41.59%) and 5 (4.42%) cases, and severe anxiety in 38 (33.63%) and 2 (1.77%) cases. There was a statistical difference between the percentage of anxiety symptoms in the FBD group and the healthy control group (*χ*^2^ = 127.163, *p* < 0.001).

In the FBD group compared to the healthy control group, no depression was detected in 12 (10.62%) and 98 (86.73%) cases, mild depression in 20 (17.70%) and 8 (7.08%) cases, moderate depression in 48 (42.48%) and 3 (2.65%) cases, and severe depression in 33 (29.20%) and 4 (3.54%) cases. There was a statistical difference between the percentage of depressive symptoms in the FBD group and the healthy control group (*χ*^2^ = 134.815, *p* < 0.001).

**Figure 9:**
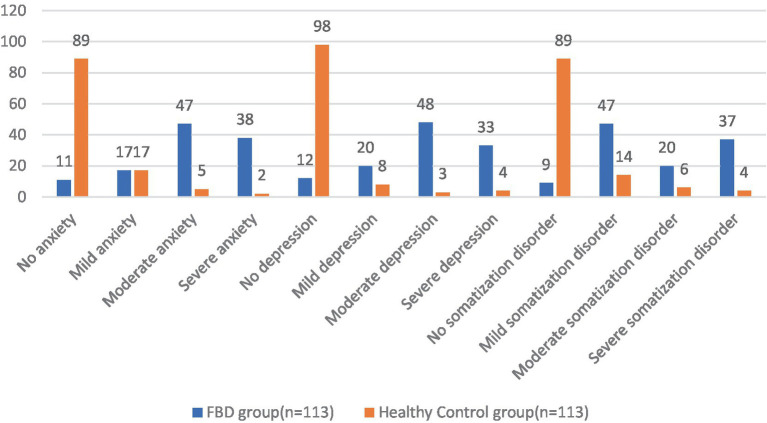
Before treatment, the severity of anxiety, depression and somatization disorder in the FBD group and the healthy control group.

In the FBD group compared to the healthy control group, no somatization disorder was detected in 9 (7.96%) and 89 (78.76%) cases, mild somatization disorder in 47 (41.59%) and 14 (12.39%) cases, moderate somatization disorder in 20 (17.70%) and 6 (5.31%) cases, and severe somatization disorder in 37 (32.74%) and 4 (3.54%) cases. There was a statistical difference between the percentage of somatization disorders in the FBD group and the healthy control group (*χ*^2^ = 117.258, *p* < 0.001) (Table 4 and Figure 9).

**Table 4:** Comparison of anxiety, depression, and somatization disorder between the FBD group and healthy control group before treatment.

	FBD group (*n* = 113)	Healthy control group (*n* = 113)	χ″ ϖαλυε	*p* value
SAS			127.163	<0.001
No	11 (9.73%)	89 (78.76%)		
Mild	17 (15.04%)	17 (15.04%)		
Moderate	47 (41.59%)	5 (4.42%)		
Severe	38 (33.63%)	2 (1.77%)		
SDS			134.815	<0.001
No	12 (10.62%)	98 (86.73%)		
Mild	20 (17.70%)	8 (7.08%)		
Moderate	48 (42.48%)	3 (2.65%)		
Severe	33 (29.20%)	4 (3.54%)		
SSS			117.258	<0.001
No	9 (7.96%)	89 (78.76%)		
Mild	47 (41.59%)	14 (12.39%)		
Moderate	20 (17.70%)	6 (5.31%)		
Severe	37 (32.74%)	4 (3.54%)		

### 3.6 Results of multi-factor linear regression analysis for each scale in the FBD group

A multi-factor linear regression equation was constructed incorporating gender, age, monthly household income, smoking history, drinking history, daily activity, and sleep quality.

The results of the study found statistical differences in the effects of different genders (female than male), different monthly household incomes, whether or not they smoked and drank alcohol, different daily activity levels, and different sleep quality on chronic constipation severity scores. However, there was no statistically significant difference in the effect of different ages (years) on chronic constipation severity scores (Table 5).

**Table 5:** Results of multi-factor linear regression analysis of the severity of chronic constipation.

Variants	*b* value	SE	*t* value	*p* value
Female*	1.578	0.782	2.019	0.045
Age	0.046	0.023	1.967	0.05
Monthly incomes	3.001	0.983	3.053	0.003
Smoking history	2.387	0.726	3.288	0.001
Drinking history	2.028	0.726	2.793	0.006
Daily activity	−2.867	0.455	−6.296	<0.001
Sleep quality	−2.599	0.589	−4.409	<0.001

Adjusted *R*^2^ = 0.344 *Male as control group.

The results of the study found statistical differences in the effects of different genders (female than male), ages (years), different monthly household incomes, whether or not they smoked and drank alcohol, different daily activity levels, and different sleep quality on anxiety severity scores (Table 6).

**Table 6:** Results of multi-factor linear regression analysis of anxiety severity.

Variants	*b* value	SE	*t* value	*p* value
Female*	6.398	1.979	3.234	0.001
Age	0.183	0.059	3.097	0.002
Monthly incomes	6.91	2.488	2.777	0.006
Smoking history	6.077	1.838	3.307	0.001
Drinking history	3.742	1.838	2.036	0.043
Daily activity	−6.781	1.153	−5.884	<0.001
Sleep quality	−3.241	1.492	−2.172	0.031

Adjusted *R*^2^ = 0.337 *Male as control group.

The results of the study found statistical differences in the effects of different genders (female than male), ages (years), different monthly household incomes, whether or not they smoked and drank alcohol, different daily activity levels, and different sleep quality on depression severity scores (Table 7).

**Table 7:** Results of multi-factor linear regression analysis of depression severity.

Variants	*b* value	SE	*t* value	*p* value
Female*	5.287	1.828	2.893	0.004
Age	0.111	0.055	2.025	0.044
Monthly incomes	7.263	2.298	3.16	0.002
Smoking history	5.514	1.698	3.248	0.001
Drinking history	4.5	1.698	2.651	0.009
Daily activity	−6.734	1.065	−6.325	<0.001
Sleep quality	−3.285	1.378	−2.384	0.018

Adjusted *R*^2^ = 0.307 *Male as control group.

The results of the study found statistical differences in the effects of different genders (female than male), ages (years), different monthly household incomes, whether or not they smoked, and different daily activity levels on somatization disorder severity scores. However, there was no statistically significant difference in the effect of drinking alcohol or not and different sleep quality on somatization disorder severity scores (Table 8).

**Table 8:** Results of multi-factor linear regression analysis of the severity of somatization symptoms.

Variants	*b* value	SE	*t* value	*p* value
Female*	2.411	1.193	2.02	0.045
Age	0.078	0.036	2.18	0.03
Monthly incomes	3.482	1.501	2.32	0.021
Smoking history	3.677	1.108	3.318	0.001
Drinking history	1.292	1.108	1.166	0.245
Daily activity	−3.280	0.695	−4.718	<0.001
Sleep quality	−1.532	0.9	−1.703	0.09

Adjusted *R*^2^ = 0.218 *Male as control group.

### 3.7 Analysis of the CSS, SAS, SDS, and SSS scale scores in the FBD treatment group and the FBD control group before the treatment

Before receiving rTMS treatment, the median chronic constipation severity score was 19.00 (17.00, 22.00) in the FBD treatment group and 19.50 (17.00, 22.00) in the FBD control group. There was no statistical difference between the overall chronic constipation severity scores of the two groups (*z* = 0.590, *p* = 0.555).

The median anxiety self-rating scale score was 66.00 (55.00, 68.00) in the FBD treatment group and 64.00 (58.00, 70.50) in the FBD control group. There was no statistical difference between the overall anxiety self-rating scale scores of the two groups (*z* = 0.125, *p* = 0.901).

The median depression self-rating scale score was 68.00 (61.00, 72.00) in the FBD treatment group and 63.50 (55.50, 70.25) in the FBD control group. There was no statistical difference between the overall depression self-rating scale scores of the two groups (*z* = 1.711, *p* = 0.087).

**Figure 10:**
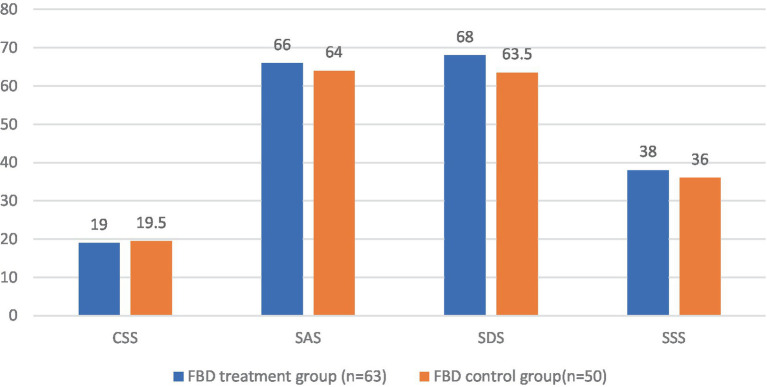
The CSS, SAS, SDS, and SSS scale scores in the FBD treatment group and the FBD control group before the treatment.

The median somatization symptom self-assessment scale score was 38.00 (34.00, 43.00) in the FBD treatment group and 36.00 (32.75, 40.00) in the FBD control group. There was no statistical difference between the overall somatization symptom self-assessment scale scores of the two groups (*z* = 1.557, *p* = 0.119) (Table 9 and Figure 10).

**Table 9:** Analysis of the CSS, SAS, SDS, and SSS scale scores in the FBD treatment group and the FBD control group before the treatment.

	FBD treatment group (*n* = 63)	FBD control group (*n* = 50)	*Z* value	*p* value
CSS	19.00 (17.00, 22.00)	19.50 (17.00, 22.00)	0.590	0.555
SAS	66.00 (55.00, 68.00)	64.00 (58.00, 70.50)	0.125	0.901
SDS	68.00 (61.00, 72.00)	63.50 (55.50, 70.25)	1.711	0.087
SSS	38.00 (34.00, 43.00)	36.00 (32.75, 40.00)	1.557	0.119

### 3.8 Analysis of the CSS, SAS, SDS, and SSS scale scores in the FBD treatment group and the FBD control group after the treatment

After receiving rTMS treatment, the median chronic constipation severity score was 8.00 (7.00, 8.00) in the FBD treatment group and 20.00 (16.00, 21.00) in the FBD control group. There was a statistical difference between the overall chronic constipation severity scores of the two groups (*z* = 9.158, *p* < 0.001).

The median anxiety self-rating scale score was 40.00 (38.00, 47.00) in the FBD treatment group and 63.00 (56.00, 69.25) in the FBD control group. There was a statistical difference between the overall anxiety self-rating scale scores of the two groups (*z* = 8.632, *p* < 0.001).

The median depression self-rating scale score was 43.00 (40.00, 48.00) in the FBD treatment group and 61.50 (54.00, 70.25) in the FBD control group. There was a statistical difference between the overall depression self-rating scale scores of the two groups (*z* = 8.214, *p* < 0.001).

**Figure 11:**
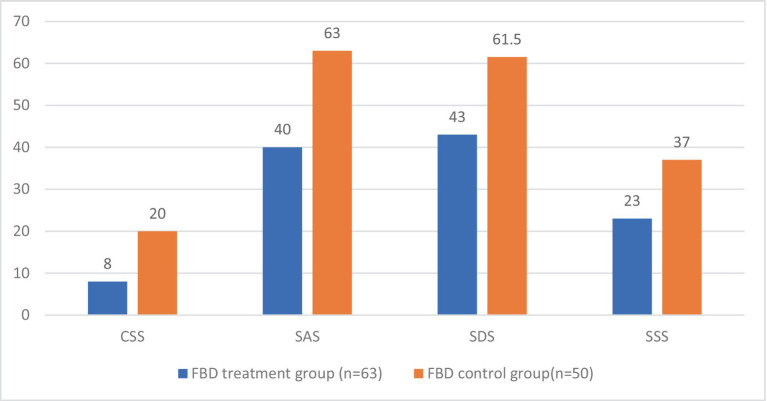
The CSS, SAS, SDS, and SSS scale scores in the FBD treatment group and the FBD control group after the treatment.

The median somatization symptom self-rating scale score was 23.00 (18.00, 29.00) in the FBD treatment group and 37.00 (31.00, 40.25) in the FBD control group. There was a statistical difference between the overall somatization symptom self-rating scale scores of the two groups (*z* = 6.993, *p* < 0.001) (Table 10 and Figure 11).

**Table 10:** Analysis of the CSS, SAS, SDS, and SSS scale scores in the FBD treatment group and the FBD control group after the treatment.

	FBD treatment group (*n* = 63)	FBD control group (*n* = 50)	*Z* value	*p* value
CSS	8.00 (7.00, 8.00)	20.00 (16.00, 21.00)	9.158	<0.001
SAS	40.00 (38.00, 47.00)	63.00 (56.00, 69.25)	8.632	<0.001
SDS	43.00 (40.00, 48.00)	61.50 (54.00, 70.25)	8.214	<0.001
SSS	23.00 (18.00, 29.00)	37.00 (31.00, 40.25)	6.993	<0.001

### 3.9 Analysis of the difference in the CSS, SAS, SDS, and SSS scale scores between the FBD treatment group and the FBD control group before and after treatment

The difference in chronic constipation severity scores before and after treatment was 11.63 ± 3.31 in the FBD treatment group and 0.74 ± 3.81 in the FBD control group. The difference in chronic constipation severity scores before and after treatment between the two groups was statistically significant (difference 10.89, 95% CI 9.57–12.22, *t* = 16.254, *p* < 0.001).

The difference in anxiety self-rating scale scores before and after treatment was 21.29 ± 9.35 in the FBD treatment group and 1.06 ± 8.31 in the FBD control group. The difference in anxiety self-rating scale scores before and after treatment between the two groups was statistically significant (difference 20.23, 95% CI 16.88–23.57, *t* = 11.992, *p* < 0.001).

The difference in depression self-rating scale scores before and after treatment was 22.37 ± 10.94 in the FBD treatment group and 1.18 ± 10.70 in the FBD control group. The difference in depression self-rating scale scores before and after treatment between the two groups was statistically significant (difference 21.19, 95% CI 17.12–25.25, *t* = 10.325, *p* < 0.001).

The difference in somatization symptom self-rating scale scores before and after treatment was 14.06 ± 11.21 in the FBD treatment group and 0.42 ± 7.32 in the FBD control group. The difference in somatization symptom self-rating scale scores before and after treatment between the two groups was statistically significant (difference 13.64, 95% CI 10.00–17.28, *t* = 7.438, *p* < 0.001) (Table 11).

**Table 11:** Analysis of the difference in the CSS, SAS, SDS, and SSS scale scores between the FBD treatment group and the FBD control group before and after treatment.

	FBD treatment group (*n* = 63)	FBD control group (*n* = 50)	Difference and 95% CI	*t* value	*p* value
CSS	11.63 ± 3.31	0.74 ± 3.81	10.89 (9.57–12.22)	16.254	<0.001
SAS	21.29 ± 9.35	1.06 ± 8.31	20.23 (16.88–23.57)	11.992	<0.001
SDS	22.37 ± 10.94	1.18 ± 10.70	21.19 (17.12–25.25)	10.325	<0.001
SSS	14.06 ± 11.21	0.42 ± 7.32	13.64 (10.00–17.28)	7.438	<0.001

### 3.10 Analysis of the CSS, SAS, SDS, and SSS scale scores in the FBD treatment group before and after rTMS treatment

Patients in the FBD treatment group had a chronic constipation severity score of 19.35 ± 2.94 before rTMS treatment and 7.71 ± 1.37 after rTMS treatment. There was a statistical difference in the overall mean chronic constipation severity score before and after rTMS treatment (difference 11.635, 95% CI 10.802–12.468, *t* = 27.914, *p* < 0.001).

Patients in the FBD treatment group had anxiety self-rating scale scores of 62.98 ± 9.68 before rTMS treatment and 41.70 ± 6.57 after rTMS treatment. There was a statistical difference in the overall mean of anxiety self-rating scale scores before and after rTMS treatment (difference 21.286, 95% CI 18.931–23.641, *t* = 18.068, *p* < 0.001).

Patients in the FBD treatment group had depression self-rating scale scores of 66.11 ± 8.60 before rTMS treatment and 43.75 ± 6.85 after rTMS treatment. There was a statistical difference in the overall mean of depression self-rating scale scores before and after rTMS treatment (difference 22.365, 95% CI 19.611–25.120, *t* = 16.231, *p* < 0.001).

**Figure 12:**
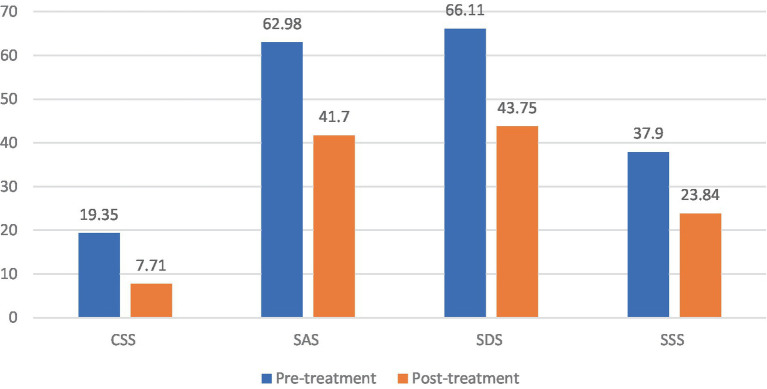
The CSS, SAS, SDS, and SSS scale scores in the FBD treatment group before and after rTMS treatment.

Patients in the FBD treatment group had symptom self-rating scales of 37.90 ± 7.24 before rTMS treatment and 23.84 ± 6.57 after rTMS treatment. There was a statistical difference in the overall mean of symptom self-rating scales before and after rTMS treatment (difference 14.063, 95%CI 11.241–16.886, *t* = 9.960, *p* < 0.001) (Table 12 and Figure 12).

**Table 12:** Analysis of the CSS, SAS, SDS, and SSS scale scores in the FBD treatment group before and after rTMS treatment.

	Pre-treatment	Post-treatment	Difference and 95% CI	*t* value	*p* value
CSS	19.35 ± 2.94	7.71 ± 1.37	11.635 (10.802–12.468)	27.914	<0.001
SAS	62.98 ± 9.68	41.70 ± 6.57	21.286 (18.931–23.641)	18.068	<0.001
SDS	66.11 ± 8.60	43.75 ± 6.85	22.365 (19.611–25.120)	16.231	<0.001
SSS	37.90 ± 7.24	23.84 ± 6.57	14.063 (11.241–16.886)	9.960	<0.001

### 3.11 Effects of rTMS on anxiety, depression, and somatization symptoms in patients with FBD

Before rTMS treatment, there were 12 patients (19.05%) with no anxiety state, 5 patients (7.94%) with mild anxiety, 33 patients (52.38%) with moderate anxiety, and 13 patients (20.63%) with severe anxiety among the 63 patients with FBD. After rTMS treatment, there were 56 patients (88.89%) with no anxiety state, 7 patients (11.11%) with mild anxiety, and no patients suffering from moderate or severe anxiety. There was a statistically significant difference between the percentage of anxiety symptoms in patients with FBD before and after rTMS treatment (*χ*^2^ = 74.804, *p* < 0.001).

There were 9 patients (14.29%) with no depressive state, 8 patients (12.70%) with mild depression, 31 patients (49.21%) with moderate depression, and 15 patients (23.81%) with major depression among the 63 patients with FBD. After rTMS treatment, there were 58 patients (92.06%) with no depressive state, 5 patients (7.94%) with mild depression, and no patients with moderate or severe depression. There was a statistically significant difference between the percentage of depressive symptoms in patients with FBD before and after rTMS treatment (*χ*^2^ = 82.528, *p* < 0.001).

**Figure 13:**
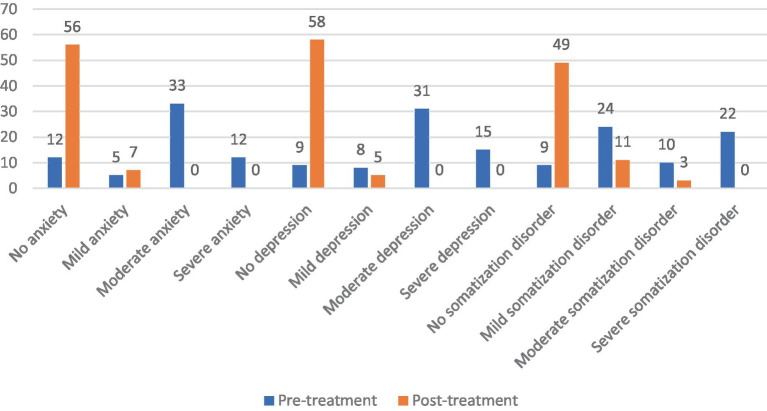
Severity of anxiety, depression and somatization disorder in the FBD group before and after treatment.

There were 7 patients (11.11%) with no somatization disorder, 24 patients (38.10%) with mild somatization disorder, 10 patients (15.87%) with moderate somatization disorder, and 22 patients (34.92%) with severe somatization disorder among the 63 patients with FBD. After rTMS treatment, there were 49 patients (77.78%) with no somatization disorder, 11 patients (17.46%) with mild somatization disorder, 3 patients (4.76%) with moderate somatization disorder, and no patients with severe somatization disorder. There was a statistically significant difference between the percentage of somatization disorders in patients with FBD before and after rTMS treatment (*χ*^2^ = 62.098, *p* < 0.001) (Table 13 and Figure 13).

**Table 13:** Effects of rTMS on anxiety, depression, and somatization symptoms in patients with FBD.

	Pre-treatment	Post-treatment	χ″ϖαλυε	*p* value
SAS			74.804	<0.001
No	12 (19.05%)	56 (88.89%)		
Mild	5 (7.94%)	7 (11.11%)		
Moderate	33 (52.38%)	0 (0)		
Severe	13 (20.63%)	0 (0)		
SDS			82.528	<0.001
No	9 (14.29%)	58 (92.06%)		
Mild	8 (12.70%)	5 (7.94%)		
Moderate	31 (49.21%)	0 (0)		
Severe	15 (23.81%)	0 (0)		
SSS			62.098	<0.001
No	7 (11.11%)	49 (77.78%)		
Mild	24 (38.10%)	11 (17.46%)		
Moderate	10 (15.87%)	3 (4.76%)		
Severe	22 (34.92%)	0 (0)		

### 3.12 Analysis of the CSS, SAS, SDS, and SSS scale scores before rTMS treatment in the FBD treatment group with different sessions of TMS

The pre-treatment chronic constipation severity scores were 19.94 ± 3.03 and 18.74 ± 2.76 in the FBD treatment group (rTMS <200 sessions) and the FBD treatment group (rTMS ≥200 sessions). There was no statistical difference between the overall mean chronic constipation severity score between the two groups (difference 1.196, 95% CI −0.264–2.655, *t* = 1.638, *p* = 0.107).

The pre-treatment anxiety self-rating scale scores were 63.63 ± 8.88 and 62.32 ± 10.54 in the FBD treatment group (rTMS <200 sessions) and FBD treatment group (rTMS ≥200 sessions). There was no statistical difference between the overall means of anxiety self-rating scale scores in the two groups (difference 1.302, 95% CI −3.602–6.207, *t* = 0.531, *p* = 0.597).

The pre-treatment depression self-rating scale scores were 64.78 ± 5.91 and 67.48 ± 10.62 in the FBD treatment group (rTMS <200 sessions) and FBD treatment group (rTMS ≥200 sessions). There was no statistical difference between the overall means of the depression self-rating scale scores of the two groups (difference − 2.073, 95% CI −7.079–1.674, *t* = −1.243, *p* = 0.220).

**Figure 14:**
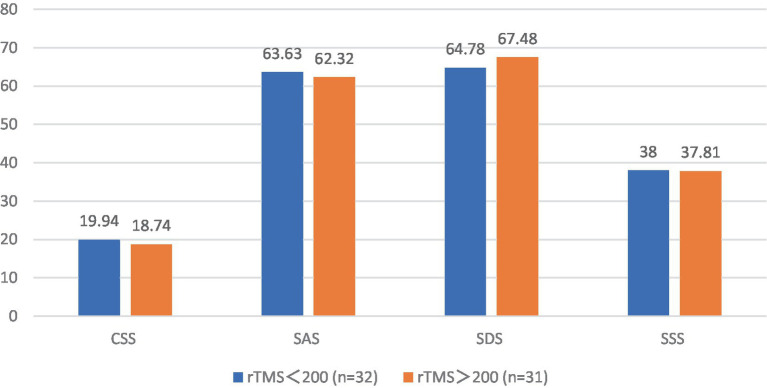
The CSS, SAS, SDS, and SSS scale scores before rTMS treatment in the FBD treatment group with different sessions of rTMS.

The pre-treatment somatization symptom self-rating scale scores were 38.00 ± 7.25 and 37.81 ± 7.36 in the FBD treatment group (rTMS <200 sessions) and the FBD treatment group (rTMS ≥200 sessions). There was no statistical difference between the overall means of the somatization symptom self-rating scale scores of the two groups (difference 0.194, 95% CI −3.486–3.873, *t* = 0.105, *p* = 0.917) (Table 14 and Figure 14).

**Table 14:** Analysis of the CSS, SAS, SDS, and SSS scale scores before rTMS treatment in the FBD treatment group with different sessions of rTMS.

	rTMS < 200 (*n* = 32)	rTMS > 200 (*n* = 31)	Difference and 95% CI	*t* value	*p* value
CSS	19.94 ± 3.03	18.74 ± 2.76	1.196 (−0.264–2.655)	1.638	0.107
SAS	63.63 ± 8.88	62.32 ± 10.54	1.302 (−3.602–6.207)	0.531	0.597
SDS	64.78 ± 5.91	67.48 ± 10.62	−2.073 (−7.079–1.674)	−1.243	0.220
SSS	38.00 ± 7.25	37.81 ± 7.36	0.194 (−3.486–3.873)	0.105	0.917

### 3.13 Effects of different sessions of TMS on patients with FBD

The post-treatment chronic constipation severity scores were 7.63 ± 1.52 and 6.29 ± 1.16 in the FBD treatment group (rTMS <200 sessions) and the FBD treatment group (rTMS ≥200 sessions). There was a statistical difference between the overall means of chronic constipation severity scores in the two groups (difference 1.335, 95% CI 0.652–2.017, *t* = 3.911, *p* < 0.001).

The post-treatment anxiety self-rating scale scores were 44.16 ± 7.76 and 39.16 ± 3.74 in the FBD treatment group (rTMS <200 sessions) and the FBD treatment group (rTMS ≥ 200 sessions). There was a statistical difference between the overall means of the anxiety self-rating scale scores of the two groups (difference 4.995, 95% CI 1.920–8.070, *t* = 3.272, *p* = 0.002).

The post-treatment depression self-rating scale scores were 46.84 ± 5.93 and 40.55 ± 6.32 in the FBD treatment group (rTMS < 200 sessions) and the FBD treatment group (rTMS ≥ 200 sessions). There was a statistically significant difference between the overall means of the depression self-rating scale scores of the two groups (difference 1.543, 95% CI 3.209–9.382, *t* = 4.079, *p* < 0.001).

**Figure 15:**
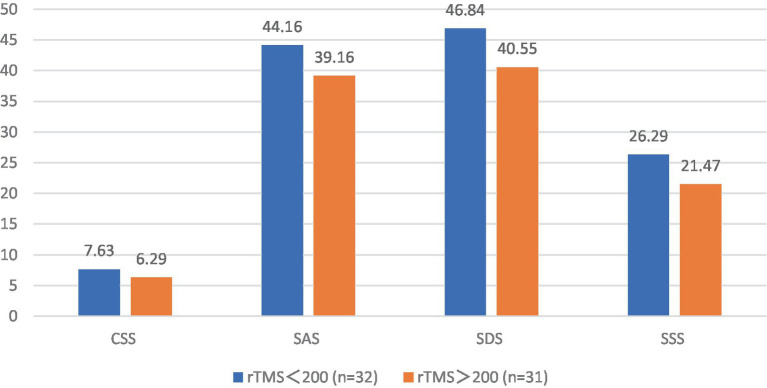
The CSS, SAS, SDS, and SSS scale scores after rTMS treatment in the FBD treatment group with different sessions of rTMS.

The post-treatment somatization symptom self-rating scale scores were 26.29 ± 6.96 and 21.47 ± 5.26 in the FBD treatment group (rTMS <200 sessions) and the FBD treatment group (rTMS ≥200 sessions). There was a statistically significant difference between the overall means of the somatization symptom self-rating scale scores of the two groups (difference 4.822, 95% CI 1.720–7.923, *t* = 3.109, *p* = 0.003) (Table 15 and Figure 15).

**Table 15:** Effects of different sessions of rTMS on patients with FBD.

	rTMS < 200 (*n* = 32)	rTMS > 200 (*n* = 31)	Difference and 95% CI	*t* value	*p* value
CSS	7.63 ± 1.52	6.29 ± 1.16	1.335 (0.652–2.017)	3.911	<0.001
SAS	44.16 ± 7.76	39.16 ± 3.74	4.995 (1.920–8.070)	3.272	0.002
SDS	46.84 ± 5.93	40.55 ± 6.32	1.543 (3.209–9.382)	4.079	<0.001
SSS	26.29 ± 6.96	21.47 ± 5.26	4.822 (1.720–7.923)	3.109	0.003

## 4 Discussion

FBD is a pervasive gastrointestinal condition that imposes a substantial burden on healthcare systems worldwide and negatively impacts patients’ quality of life. A study that spanned 33 countries across six continents found that over 40% of the global population suffers from functional gastrointestinal disorders (39). The primary symptoms of FBD include abdominal pain, bloating, abdominal distension, abdominal discomfort, diarrhea, constipation, or alternating diarrhea and constipation, with each subtype of FBD manifesting a combination of these symptoms (40). The disease is recurrent, often without organic lesions, and is characterized by dysfunction of intestinal sensory, secretory, and motor functions, as well as dysbiosis of the intestinal microbiota (2, 3). Chronic gastrointestinal discomfort poses significant challenges for patients, and over time, many develop psychological disorders and mental symptoms. Persistent anxiety and depression can impact the hypothalamus, vegetative nerves, and inhibit peripheral autonomic nerves, exacerbating intestinal discomfort and increasing the treatment burden (6).

The etiology of FBD remains unclear, with studies suggesting that the condition arises from a complex interplay of physiological, psychological, genetic, social, and early-life factors (4). With the introduction of Rome IV criteria, the brain-gut axis has been recognized as a foundational element in the pathophysiology of functional bowel disease (7). Increasing evidence supports the theory that the progression of functional bowel disease occurs in three stages: stage-I gastrointestinal dysmotility, stage-II visceral hypersensitivity, and stage-III bidirectional brain-gut axis dysfunction (8, 9). Persistent gastrointestinal symptoms have a profound impact on patients’ physical and mental well-being. Over time, many patients develop symptoms such as anxiety, depression, and somatization disorders (5). Therefore, diagnosing functional bowel disease should encompass not only gastrointestinal symptoms but also mental symptoms and somatization disorders.

The use of functional brain imaging enables the identification and localization of the *in vivo* activity of the human brain with a high degree of anatomical precision. There is emerging evidence that the central nervous system processing of noxious visceral stimuli may be abnormal in patients with irritable bowel syndrome. Although the results were variable, all involved the anterior cingulate cortex and prefrontal cortical regions (41). Increased local cerebral blood flow in the anterior cingulate cortex, insula cortex activates hippocampal, thalamic, and hypothalamic responses to visceral stimuli (42). In a study of brain responses to visceral stimulation, Mayer et al. (43) found that the most common areas of brain activity were the insula and anterior cingulate cortex, followed by the primary sensory cortex, prefrontal cortex, posterior parietal cortex, and thalamus. In their study, Tillisch et al. (44) found that brain differences between patients with irritable bowel syndrome and healthy controls were mainly in the insula, thalamus, anterior cingulate cortex, amygdala, middle cingulate cortex, midbrain, postcentral gyrus, precentral gyrus, prefrontal cortex, and inferior parietal lobule.

Brain stimulation through TMS is a non-invasive technique that modulates brain activity using a magnetic field to induce an electric current in the brain. The magnetic field has a robust penetrating capability within the brain (14). Generally, single-pulse and double-pulse TMS are used to explore brain function, while rTMS aims to induce changes in brain activity that persist beyond the stimulation period (15). The changes in brain activity depend on the excitability of the stimulated area, as well as the intensity and frequency of the stimulation. rTMS induces changes in distal brain regions, with low-frequency (<1HZ) rTMS having an inhibitory effect and high-frequency (> 5HZ) rTMS exerting an excitatory effect (16). Brain stimulation has emerged as a potential therapeutic modality for FBD, leveraging the influence of the brain-gut axis in the disease process.

Among the 113 patients with functional bowel disease in this study, the incidence of females (68.14%) was significantly higher than the incidence of males (31.86%). The average age of patients with functional bowel disease was 65.45 ± 14.64 years, with female patients concentrated in the age group of 60–69 years and male patients in the age group of 60–79 years. Most studies have shown that women are at higher risk for functional bowel disease compared to men (45). The reason for the higher incidence of middle-aged and elderly women may be related to their physiological characteristics, decreased hormone levels, and greater pressure in society (46).

In recent years, many studies have shown that many social factors such as excessive life and financial stress, emotional problems, and family problems can have a negative impact on individuals (47). Negative life events can cause individuals to suffer from poor physical and mental health. If these negative emotions are not improved, they may become a risk factor for the development of functional bowel disease. For a family, financial stress is an important factor that can have long-term negative effects. There were 61 cases (53.98%) with poor monthly household income in the functional bowel disease group and 38 cases (33.63%) with poor monthly household income in the healthy control group. Poor family income has brought enormous stress on the patient’s body, mind, and life, which can lead to excessive mental stress and thus induce the occurrence of functional bowel disease (48). Excessive stress can pose a great threat to the homeostasis of the body’s internal environment (49). The brain-gut axis is a bidirectional regulatory axis linking the brain and the gastrointestinal tract through a neuro-immune-endocrine network. The central nervous system, autonomic nervous system, and enteric nervous system are the main pathways of the brain-gut axis (10, 11). Long-term negative events can affect the regulation of neurotransmitters in the central nervous system, which in turn can cause gastrointestinal discomfort through the immunoendocrine network.

In this study, the daily activity of the functional bowel disease group was significantly lower than that of the healthy control group. There was a statistically significant difference between the two groups, *p* < 0.001. With the rapid development of society and the acceleration of the pace of life, people’s social pressure has also increased, and more and more people tend to stay at home to relieve the fatigue of life. With the rapid development and popularity of electronic products, more and more people are using electronic products as a way to relax. People stay in a space without contact with the outside world for a long time. Over time, it will not only have a negative impact on mental status, but also affect the imbalance of intestinal flora if they do not engage in outdoor activities for a long time (50). There is growing evidence of a link between the gut microbiota and functional bowel disease, with dysbiosis of the gut flora leading to changes in gases and metabolites that interact with the intestinal wall and in turn lead to symptoms in the gastrointestinal tract (51). Several recent studies have shown that outdoor activity can regulate gut microbiota and reduce intestinal inflammation (52).

In this study, it was found that the number of people dissatisfied with sleep quality was significantly higher in the functional bowel disease group than in the healthy control group. There was a statistically significant difference between the two groups, *p* < 0.001. Previous clinical investigations have found a strong positive correlation between the severity of functional bowel disease and sleep disturbance (53). Chronic sleep disorders can cause abnormal movements of the gastrointestinal tract, and visceral hypersensitivity reactions are also associated with poor sleep quality (54).

The relationship between functional bowel disease and psychological disorders has received widespread attention (55). It is estimated that at least half of people with irritable bowel syndrome suffer from a psychiatric disorder (56). Among them, anxiety disorders are the most common, with a prevalence of about 30–50%, followed by depression, with a prevalence of about 25–30% (57). In this study, among 113 patients with functional enteropathy, 17 (15.04%) had mild anxiety, 47 (41.59%) had moderate anxiety, and 38 (33.63%) had severe anxiety. There were 20 (17.70%) with mild depression, 48 (42.48%) with moderate depression, and 33 (29.20%) with severe depression. There were 47 (41.59%) with mild somatization disorder, 48 (17.70%) with moderate somatization disorder, and 33 (32.74%) with severe somatization disorder. The number of people with anxiety, depression, and somatization disorders in the functional bowel disease group was significantly higher than the number of healthy controls, and the results were statistically different, *p* < 0.001. In addition, patients in the functional bowel disease group had significantly higher CSS, SAS, SDS, and SSS scale scores than the healthy control group scores, and the results were statistically different, *p* < 0.001. In the biopsychosocial model, multiple biological, psychological, and sociological factors have been considered relevant to the pathogenesis of functional bowel disease (58). There is a close connection between our gut and brain in the form of a brain-gut axis, and this close connection has become a hot topic in the current study of functional bowel disease. Several studies have shown that emotions, stress, and psychological factors are closely related to the pathogenesis of functional bowel disease (59–62). There is a clear correlation between functional bowel disease and common psychological disorders (63). Patients with persistent functional bowel disease not only suffer from the discomfort of gastrointestinal symptoms but also from mental and psychological disorders, so psychological guidance and mental regulation also play a role in the treatment of functional bowel disease.

In this study, a multifactorial linear regression equation was constructed incorporating gender, age, monthly household income, history of smoking, history of alcohol consumption, daily activity, and sleep quality. It was found that different genders (female than male), ages (years), monthly household income, histories of smoking and alcohol consumption, daily activity, and sleep quality affected constipation, anxiety, depression, and somatization disorder symptoms in patients with functional bowel disease.

In the past, the majority of treatment options for functional bowel disease were medication or symptomatic treatment. Irritable bowel syndrome is one of the common types of functional bowel disease, and the main treatment goal is to relieve intestinal discomfort and abdominal pain. The effect of using pain relief therapy and placebo therapy is not significant. Pain relief therapy is mostly treated with opioids, which can cause adverse effects such as constipation over time. The placebo itself may have a biological analgesic response, causing adverse effects that may be more pronounced than pain relief. These treatment options only relieve symptoms but do not address the root cause of functional bowel disease.

In this study, chronic constipation severity scores, anxiety self-rating scale scores, depression self-rating scale scores, and somatization symptom self-rating scale scores were significantly lower in the FBD treatment group than in the FBD control group after receiving rTMS treatment. The results were statistically different, *p* < 0.001. Through the pre-post comparison, it was found that the chronic constipation severity scores, anxiety self-rating scale scores, depression self-rating scale scores, and somatization symptom self-rating scale scores in the FBD treatment group were significantly lower than the pre-treatment scores after receiving rTMS treatment. The results were statistically different, *p* < 0.001. And after treatment, the number of anxiety, depression, and somatization disorders was significantly reduced and the severity was also significantly decreased. The results were statistically different, *p* < 0.001. After receiving rTMS treatment, the chronic constipation severity score, anxiety self-rating scale score, depression self-rating scale score, and somatization symptom self-rating scale score decreased more in the ‘FBD treatment group (rTMS ≥200 sessions)’ than in the ‘FBD treatment group (rTMS <200 sessions)’. The results were statistically different, *p* < 0.001. The application of rTMS can lead to changes in both the local area being stimulated and distal areas connected to it, leading to alterations in neural networks. As the number of rTMS sessions increases, these neural adaptations accumulate, potentially leading to more significant and sustained therapeutic effects (64). Studies have shown that patients with constipation are often accompanied by psychiatric symptoms such as anxiety and depression (30). In this study, transcranial magnetic stimulation therapy not only improved the intestinal symptoms of patients with functional bowel disease but also alleviated the patients’ psychiatric symptoms and psychological disorders. During the period of receiving rTMS treatment, all patients with functional bowel disease enrolled in the study did not experience any obvious adverse effects.

In summary, the clinical manifestations of patients with functional bowel disease are not only limited to gastrointestinal symptoms, but also often accompanied by psychiatric symptoms such as anxiety and depression. Negative events such as excessive life stress and unhealthy habits play an important role in the pathogenesis and are associated with psychological disorders in patients. These factors work together to cause persistent gastrointestinal and psychiatric symptoms in patients. Therefore, in the diagnosis and treatment of functional bowel disease, we should not only focus on the gastrointestinal symptoms of patients, but also treat their psychiatric symptoms, which is equally important. Based on the theory that the brain-gut axis plays a role in functional bowel disease, transcranial magnetic stimulation therapy breaks through the traditional method of drug treatment and symptomatic treatment, which not only improves the gastrointestinal symptoms of patients with functional bowel disease, but also relieves patients’ mental symptoms and psychological disorders.

However, we chose lower stimulation intensities compared to other studies. In our study, we chose 80% of the motor threshold as the stimulation intensity. This decision was based on our findings that functional bowel disease patients predominantly occur in individuals aged 50 and above, who may be more susceptible to adverse reactions from high-intensity stimulation. Therefore, in order to minimize the risk of potential side effects, we opted for 80% of the motor threshold as the stimulation intensity. Some patients completed fewer than 200 rTMS sessions in total. This was not part of the study design and was due to various reasons, including scheduling conflicts, voluntary withdrawal by patients who perceived improvement, and the impact of the COVID-19, which prevented some patients from being admitted for treatment.

## 5 Conclusion

We found a higher prevalence of functional bowel disease in women. Anxiety, depression, and somatization disorders were prevalent in patients with functional bowel disease. Patients with poorer household income satisfaction, lower daily activity, and poorer sleep quality had a higher likelihood to develop functional bowel disease. The concomitant symptoms of functional bowel disease (constipation, anxiety, depression, and somatization symptoms) were associated with gender, age, monthly household income, history of smoking and alcohol consumption, daily activity level, and sleep quality. rTMS has shown significant efficacy in terms of gastrointestinal symptoms, anxiety and depressive symptoms, and somatization disorders in patients with functional bowel disease. The effectiveness of rTMS treatment for patients with functional bowel disease was positively correlated with the number of treatments received. The use of rTMS for functional bowel disease has good patient compliance and acceptance, and provides rapid symptom relief with no significant adverse effects.

This study presents valuable preliminary findings on the application of rTMS in the treatment of FBD. However, we acknowledge several limitations that warrant consideration in the interpretation of our results. Firstly, the relatively small sample size may limit the generalizability of the findings to a broader population. Secondly, the study duration restricted the long-term assessment of the treatment’s efficacy.

Given these limitations, future research should aim to validate the findings of the present study with larger sample sizes, thereby enhancing the generalizability of the results. Long-term follow-up studies are crucial to evaluate the durability of the treatment effects over time. Additionally, further research could explore variations in stimulation parameters, such as frequency and intensity, to optimize the treatment protocol and improve its effectiveness.

In conclusion, while our study sheds light on the potential benefits of rTMS in managing FBD, the field would benefit from further research to confirm these findings and optimize treatment protocols. The exploration of different stimulation parameters and the conduct of long-term studies will be valuable steps in fully realizing the therapeutic potential of rTMS for FBD.

## Data availability statement

The raw data supporting the conclusions of this article will be made available by the authors, without undue reservation.

## Ethics statement

The studies involving humans were approved by the Ethics Committee of Dalian Third People’s Hospital. The studies were conducted in accordance with the local legislation and institutional requirements. Written informed consent for participation in this study was provided by the participants’ legal guardians/next of kin. Ethical approval was not required for the study involving animals in accordance with the local legislation and institutional requirements because the study does not include animals. Written informed consent was obtained from the individual(s) for the publication of any potentially identifiable images or data included in this article.

## Author contributions

GL, TL, and BJ wrote the main manuscript text. GL prepared the tables. ZF revised the manuscript. All authors reviewed the manuscript.
